# Evaluation of different serological assays for early diagnosis of leptospirosis in Martinique (French West Indies)

**DOI:** 10.1371/journal.pntd.0005678

**Published:** 2017-06-23

**Authors:** Christophe Courdurie, Yohann Le Govic, Pascale Bourhy, Dorothee Alexer, Karine Pailla, Rafaelle Theodose, Raymond Cesaire, Jacques Rosine, Patrick Hochedez, Claude Olive

**Affiliations:** 1 Department of Bacteriology and Hygiene, University Hospital of Fort-de-France, Martinique, French West Indies; 2 Department of Spirochetes Biology, Pasteur Institute, National Reference Center and WHO Collaborating Center for Leptospirosis, Paris, France; 3 Department of Virology, University Hospital of Fort-de-France, Martinique, French West Indies; 4 Interregional Epidemiology Unit, French Institute for Public Health Surveillance, Fort-de-France, Martinique, French West Indies; 5 Department of Infectious Diseases, University Hospital of Fort-de-France, Martinique, French West Indies; Oxford University Clinical Research Unit, VIET NAM

## Abstract

**Background:**

Leptospirosis is a potentially life-threatening but curable zoonosis whose prognosis depends on accurate and timely diagnosis. Because of its non-specific clinical presentation, laboratory testing is essential to confirm the diagnosis. Here, we aimed to assess the performance of two enzyme-linked immunosorbent assays (ELISAs) (ELISA Serion and ELISA-Hb Pasteur) and one immunodot (GenBio) using quantitative PCR (qPCR) as gold standard, instead of the traditional microscopic agglutination test, for the diagnosis of acute leptospirosis in an endemic area.

**Methodology/Principal findings:**

Between January 2011 and December 2012, a total of 122 patients were diagnosed with leptospirosis, as confirmed by qPCR at the University Hospital of Martinique. Among them, 103 had at least one serum sample available for analysis. Performance of each serological assay was evaluated according to days' post onset of symptoms (DPO) and local species diversity (which included *L*. *santarosai*, *L*. *interrogans*, *L*. *kirschneri*, *L*. *borgpetersenii*, *L*. *noguchii*, and *L*. *kmetyi*). Several thresholds were tested to optimize accuracy. When considering the manufacturer's threshold, the sensitivity of ELISA Serion, ELISA-Hb Pasteur and GenBio immunodot was 75%, 67% and 64%, while specificity was 92%, 98% and 100%, respectively. Moreover, the threshold optimization allowed a significant improvement in specificity for the ELISA Serion from 92% to 99% (p<0.05). During the first 5 DPO, sensitivities were 35%, 30% and 42% for ELISA Serion, ELISA-Hb Pasteur and GenBio immunodot, respectively. However, between 6─10 DPO, these sensitivities dramatically increased to reach 86%, 76% and 67%, respectively. Performances of the three assays were not affected by the species studied.

**Conclusions/Significance:**

All these serological assays showed the potential for diagnosing leptospirosis after (but not before) 6 days’ post onset of symptoms. In a high prevalence setting, where highest specificities are needed, threshold optimizing should be performed for this purpose.

## Author summary

Leptospirosis is a ubiquitous zoonosis caused by spirochetes. This infection is responsible for a wide range of symptoms mimicking many other diseases. Laboratory tests are thus essential to confirm the diagnosis. Furthermore, earlier diagnosis helps to prevent severe outcomes by a prompt initiation of antibiotic therapy. In early phase, quantitative Polymerase Chain Reaction (qPCR) was proved to be more efficient than the traditional microagglutination test (MAT), which remains the ‘gold standard’ for diagnosis of leptospirosis. However, both tests are seldom available in resource-limited settings, where leptospirosis burden is highest. To find a suitable alternative to qPCR and MAT in the early phase (i.e., within the first 2 weeks) of the disease, we compared 3 serological assays (2 ELISAs plus one immunodot) using sera collected from patients with a qPCR-confirmed diagnosis of leptospirosis. This work reveals high performances for all serological assays after (but not before) 6 days’ post onset of symptoms, regardless of the species studied. Excellent specificity, as found after threshold optimization, suggests that these tests could useful in high prevalence areas.

## Introduction

Leptospirosis is a ubiquitous zoonosis caused by pathogenic spirochetes of the genus *Leptospira*. With an estimated 1.03 million cases and 58,900 deaths occurring each year, leptospirosis represents a major threat to public health worldwide [1]. This is particularly true in the Caribbean where morbidity and mortality rates are up to 50 and 2.90 percent, respectively [2–5]. Clinical diagnosis is challenging because of the nonspecific and protean manifestations. Indeed, leptospirosis may mimic other infectious diseases commonly found in tropical climates such as dengue, Q fever, typhoid, or malaria [6–9]. Leptospirosis can also be misdiagnosed with more widely distributed illnesses including common viral infections (e.g., cold, flu and human herpesviruses infections), HIV seroconversion, primary toxoplasmosis, rickettsiosis, Hantavirus infection and brucellosis [10]. Severity ranges from relatively mild flu-like symptoms to Weil’s syndrome, a severe and potentially life-threatening form of the disease characterized by multiple organ failure, including the liver, kidneys, lungs, heart and brain [11–13]. Laboratory tests are therefore of paramount importance for a timely diagnosis (and subsequent treatment) of leptospirosis.

Human infection mainly occurs following contact with water or soil contaminated by the urine of infected animals. Leptospires enter the body through damaged skin, e.g. cuts and abrasions, or via mucous membranes. After penetration, these agents circulate in the bloodstream, with a bacteremia lasting for up to 10–15 days’ post onset of symptoms (DPO) [14]. Detectable antibodies appear in the blood about 5–10 DPO [10], and sometimes later, especially if antibiotic treatment is instituted [15].

According to WHO’s recommendations, three types of laboratory tests can be used for confirmatory diagnosis of leptospirosis: (i) isolation of *Leptospira* by culture methods, (ii) DNA detection by polymerase chain reaction (PCR), and (iii) antibody detection by microscopic agglutination test (MAT) [16,17]. MAT titer of ≥1:400 for a single or paired serum samples, as well as four-fold (or greater) increase in titers between acute and convalescent samples, are considered as positive for MAT. Most published studies comparing serological assays for the diagnosis of acute leptospirosis used MAT as gold standard; however, there are few reports arguing the capability of MAT being the reference method [18]. Indeed, in spite of its high specificity, MAT lacks sensitivity, especially during the acute phase of leptospirosis, when antibiotics are expected to have the greatest benefit [19]. Furthermore, MAT is restricted to a few reference laboratories since it requires specific equipment and highly trained staff to maintain living culture panel of leptospires (which is a risk factor of laboratory acquired infection to the technicians) as well as to analyze its results (which is somewhat subjective) [20,21].

PCR-based testing has been reported to be a useful diagnostic tool in the first week of the disease [14,22], and could thus constitute a valuable alternative to MAT during the acute phase [23]. Nevertheless, studies that employed PCR as gold standard for the comparison of different serological assays are very scarce [24]. More recently, a number of quantitative PCR (qPCR) assays have been developed to improve the diagnosis of leptospirosis [25,26]. If a qPCR-based strategy is routinely applied in our hospital, several limitations prevent its use in many other tropical areas with high disease prevalence, such as the need for specific equipment, highly trained staff and cost.

In order to provide early treatment in the course of the disease, as recommended by WHO guidance [14], it is important to identify rapid and reliable alternative assays for early phase of the disease. Hence, we aimed to assess the performance of two enzyme-linked immunosorbent assays (ELISAs) (ELISA Serion and ELISA-Hb Pasteur) and one immunodot (GenBio) using quantitative PCR (qPCR) as gold standard for the diagnosis of acute leptospirosis in Martinique. Diagnostic accuracies were determined according to days’ post onset of symptoms (DPO) and the regional species diversity [27]. Several thresholds were tested to optimize assay accuracy.

## Materials and methods

### Study design and patients

This retrospective study was conducted at the University Hospital of Martinique (which encompasses 3 sites located in Fort-de-France, La Trinité and Le Lamentin) between January 2011 and December 2012. The inclusion criteria were: (i) patients with a qPCR-confirmed diagnosis of leptospirosis, (ii) the availability of a DPO record, and (iii) the availability of at least one serum sample. At the time of admission, demographic characteristics, DPO and antibiotic prescriptions were recorded on a standardized report form. Severe leptospirosis was defined by the presence of at least one of the following criteria: shock treated with vasoactive drugs, acute renal failure requiring dialysis, internal bleeding requiring blood transfusion, respiratory insufficiency requiring mechanical ventilation, or death.

### Samples

Blood plasma samples were collected in ethylenediaminetetraacetic acid (EDTA) tubes obtained from patients with clinically suspected leptospirosis. Serum specimens were collected in plastic red-top tubes at different times during the course of the illness and stored at -20°C until testing.

To assess the specificity of the different serological tests, a panel of 121 serum samples positive for other diseases than leptospirosis (mainly primary viral infection) was selected as follows: 40 dengue sera (PCR and IgM positive or IgG and IgM positive), because dengue fever is likely the most common differential diagnosis of leptospirosis among Carribean patients; 10 anti-EBV IgM and IgG positive sera; 15 anti-CMV IgM and IgG positive sera; 4 HIV serology positive sera (HIV core protein p24 and anti-HIV-1/2 antibodies as confirmed by Western Blot); 10 anti-*Toxoplasma gondii* IgM and IgG positive sera; 20 syphilis serology positive sera (simultaneously positive for VDRL and FTA-ABS) since *Treponema pallidum* is a related spirochete; 13 rheumatoid factor-positive sera (simultaneously positive for Waaler-Rose and Latex tests) and 9 antinuclear antibody positive sera, which are known to cross-react in several serological assays.

### Quantitative PCR

Detection of leptospiral DNA was performed as previously described [28]. Briefly, after EDTA-treated blood centrifugation, DNA was extracted with the QIAamp DNA Mini Kit (Qiagen SA, Courtaboeuf, France) according to the manufacturer’s instructions. Extracts were then used to perform a SYBR green assay (Bio-Rad, Hercules, CA, USA) selective for *lfb1* gene. Amplifications were done with the LightCycler 480 Thermal Cycling System (Roche Diagnostics, Basel, Switzerland).

### Serological tests

**Serion IgM-ELISA** (Institut Virion\Serion GmbH, Würzburg, Germany) was performed following the manufacturer’s instructions and as described previously [29], with a HydroFlex^TM^ microplate washer (Tecan Trading AG, Switzerland) and a PR 3100 TSC plate reader (Bio-Rad Laboratories, CA, USA) measuring absorbance at 450 nm. This test uses crude antigens from an isolated, concentrated and partially purified extract of *Leptospira biflexa* serovar Patoc strain Patoc I (ATCC 23582), which contains genus specific epitopes for all *Leptospira* spp. Interpretation of results was as follows: anti-leptospiral IgM <15 IU/ml gives a negative result, 15–19 IU/ml gives a borderline result and ≥20 IU/ml gives a positive result.

**In-House IgM ELISA-Hb Pasteur** (National Reference Center of leptospirosis at Pasteur Institute, Paris, France) was performed as described previously [30], with the aforementioned microplate washer and reader. This test uses crude preparation of formalin-treated and boiled *Leptospira fainei* serovar Hurstbridge strain BUT 6^T^, allowing detection of *Leptospira*-specific IgM class antibodies. Titers of ≥1:400 are considered to be significant.

**GenBio IgM ImmunoDOT** (GenBio, San Diego, USA), was performed according to the manufacturer’s instructions and as described previously [31]. This test is a rapid, easy-to-use, semi-quantitative enzyme immunoassay (EIA) that specifically detects IgM antibodies against *Leptospira biflexa* (serovar Patoc I). Results are interpreted as the number of reactive dots observed: 0 to 1 for negative, 2–2.5 for borderline positive, and 3–4 for a strong positive.

### Molecular characterization

Leptospires were identified at the species level by partial sequencing of the *secY* and *rrs* genes, as previously described [27,32]. All molecular epidemiological data were stored and analyzed with Bionumerics software (Version 6.5; Applied-Maths, Belgium).

### Statistical analyses

All statistics were performed using Excel 2010 (Microsoft Corporation, Redmond, United States of America). Data were presented as percentage with 95% confidence intervals (95% CI), or median values with their interquartile range (IQR). Sensitivity (Se), specificity (Sp), positive and negative predictive values (PPV and NPV, respectively) according to prevalence, and receiver operating characteristics (ROC) [33] curves were calculated for each assay using qPCR as reference method. Assay comparison was performed by Chi-square test. Likelihood ratios (LRs) were also determined according to whether a test was positive (LRP = Se/(1-Sp)), or negative (LRN = (1-Se)/Sp) [34]. These values were further combined to calculate the diagnostic odds ratio (DOR = LRP/LRN), which quantifies test performance by combining the strengths of sensitivity and specificity, with the advantage of representing a single indicator, higher values indicating better discriminatory test performance [35].

### Ethics statement

The study was approved by the French Human Ethics Committee and declared on number 1925033v0. All patients who agreed to enroll in the study signed an informed consent. In those cases where patients were under 18 years old (legal age), informed consent was signed by the father, mother, or assigned tutor.

## Results

### Patient characteristics

From January 2011 through December 2012, a total of 122 patients were qPCR-confirmed for leptospirosis at the University Hospital of Martinique. Among them, 19 were subsequently excluded either because of lack of serum sample (n = 17) or lack of knowledge on DPO (n = 2). Finally, a total of 103 eligible patients were included in this study.

Most of these 103 patients were men (n = 91, 88%), and the median age was 49 years (IQR: 37–58). The median time between onset of symptoms and plasma sample collection—allowing qPCR-based diagnosis of leptospirosis—was 3 days (IQR: 2–5), while the first serum sample was collected about 9 days (IQR: 5–15) after the beginning of symptoms. A second serum sample was available for 38 patients (37%), with a median delay between first and second sample of 11 days (IQR: 7–15). There were 9 cases with severe disease. No deaths occurred in this consecutive series (S1 Table).

### Influence of threshold on test accuracy

Firstly, diagnostic accuracy was determined for each assay on the earliest serum sample considering manufacturers' thresholds (Table 1). The three tests showed quite similar sensitivities, but ELISA Serion was significantly less specific (Sp = 92%) than both ELISA-Hb Pasteur (Sp = 98%, p = 0.018) and GenBio immunodot (Sp = 100%, p = 0.001). To optimize the performance of each assay, different thresholds were then evaluated. The sensitivity and specificity were calculated for each threshold and each assay. ROC curves were also generated to determine the optimal cut-off value for each test (S1–S3 Appendixes). We found an optimal threshold of 35 IU/mL for ELISA Serion, 1:400 last positive dilution for ELISA-Hb Pasteur (which corresponds to the threshold that was initially determined by the Pasteur Institute) and 1 dot for GenBio immunodot. Overall accuracy was subsequently calculated with these optimized thresholds, as reported in Table 1.

**Table 1:** Comparison of three serological assays for leptospirosis diagnosis using qPCR as gold standard.

	ELISA Serion		Genbio immunodot		ELISA-Hb Pasteur
Threshold	20 UI/mL	35 IU/mL	2 dots	1 dot	1/400^*^
% Sensitivity (95% CI)	75 (66–83)	70 (61–78)	64 (54–72)	69 (59–76)	67 (57–75)
% Specificity (95% CI)	92^1^^,^^2^ (85–95)	99 (95–100)	100 (97–100)	100 (97–100)	98 (94–100)
LRP (95% CI)	9 (5–16.6)	85 (12–602)	NA	NA	40 (10–160)
LRN (95% CI)	0.27 (0.19–0.38)	0.3 (0.22–0.4)	0.36 (0.28–0.47)	0.31 (0.24–0.42)	0.34 (0.26–0.45)
DOR(95% CI)	34 (15–74)	286 (38–2143)	NA	NA	119 (28–510)

Sensitivity, Specificity, Likelyhood Ratio Positive (LRP) and Negative (LRN) and Diagnostic odds ratio (DOR), are presented for each test, before (unhighlighted) and after (gray highlighted) threshold optimization.

^*^ threshold for ELISA-Hb Pasteur remained unchanged.

^1^ significant difference between before and after optimizing threshold.

^2^ significant difference between tests.

NA, not applicable

**Fig 1:**
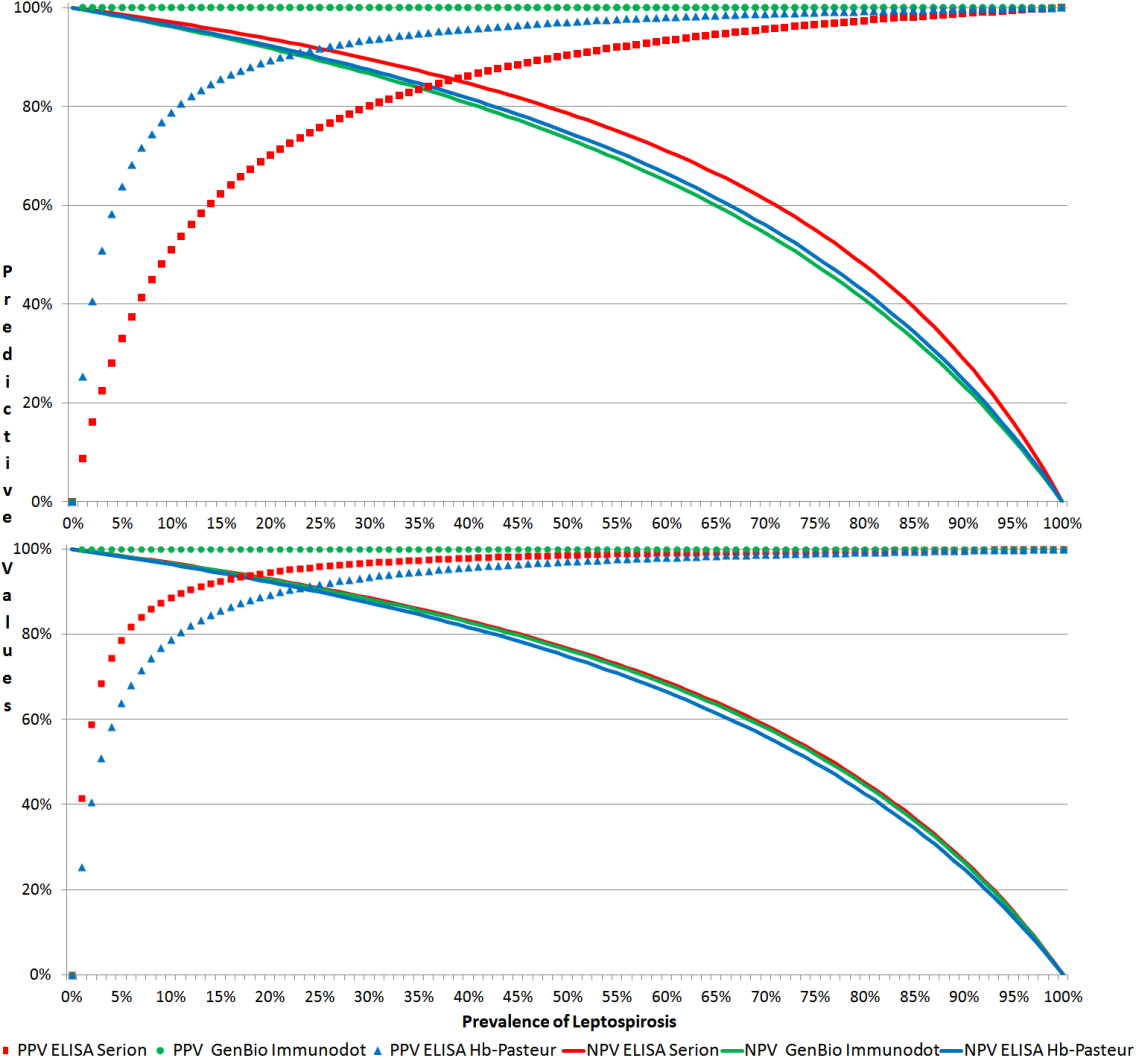
Predictive values according to estimated prevalence before (top) and after (bottom) threshold optimization.

It was not possible to determine LRP and DOR for GenBio because of its 100% specificity. Overlapping CIs revealed no marked differences between the three tests for LRP and DOR. However, the benefit of threshold optimization was detectable for predictive values in low prevalence situation, e.g. for ELISA Serion, PPV increased from 8.5% to 41% (Fig 1).

Further analysis was conducted using these optimized thresholds.

### Effect of DPO

To evaluate the influence of DPO on diagnostic accuracy, sensitivities were calculated for the three tests during the 0–5, 6–10, 11–15 and > 15 DPO intervals, as reported in Table 2. There was no difference between the sensitivity of the 3 tests, regardless of the time elapsed between the first symptoms and serum sampling. However, a statistically significant increase in sensitivity was observed between 0–5 and 6–10 DPO intervals for the two ELISAs (p<0.05), but not for the GenBio Immunodot (p = 0.11).

**Table 2:** Sensitivities of the three serological assays according to DPO.

		Test		
Number of patients	DPO	ELISA Serion	ELISA Pasteur-Hb	GenBio Immunodot
n = 40	0–5	35 (22–50)	30 (18–45)	42 (29–58)
		p<0.05	p<0.05	NS
n = 21	6–10	86 (65–95)	76 (55–89)	67 (45–83)
		NS	NS	NS
n = 16	11–15	94 (72–99)	93 (72–99)	94 (72–99)
		NS	NS	NS
n = 26	>15	92 (75–98)	96 (80–99)	92 (75–98)

n are number of cases included during each DPO interval.

Significantly different results between two consecutive DPO intervals are indicated by p ˂0.05. NS, not statistically different.

### Impact of paired sera on diagnostic performance

The median time elapsed from onset of symptoms until collecting a second sample was 14 days (IQR: 9–19). When considering the latest sample (of a paired sera), the sensitivity of all tests increased significantly, reaching 85%, 86% and 85% for ELISA Serion, ELISA Hb-Pasteur and GenBio immunodot, respectively (p<0.05).

### Accuracies and molecular characterization

**Fig 2:**
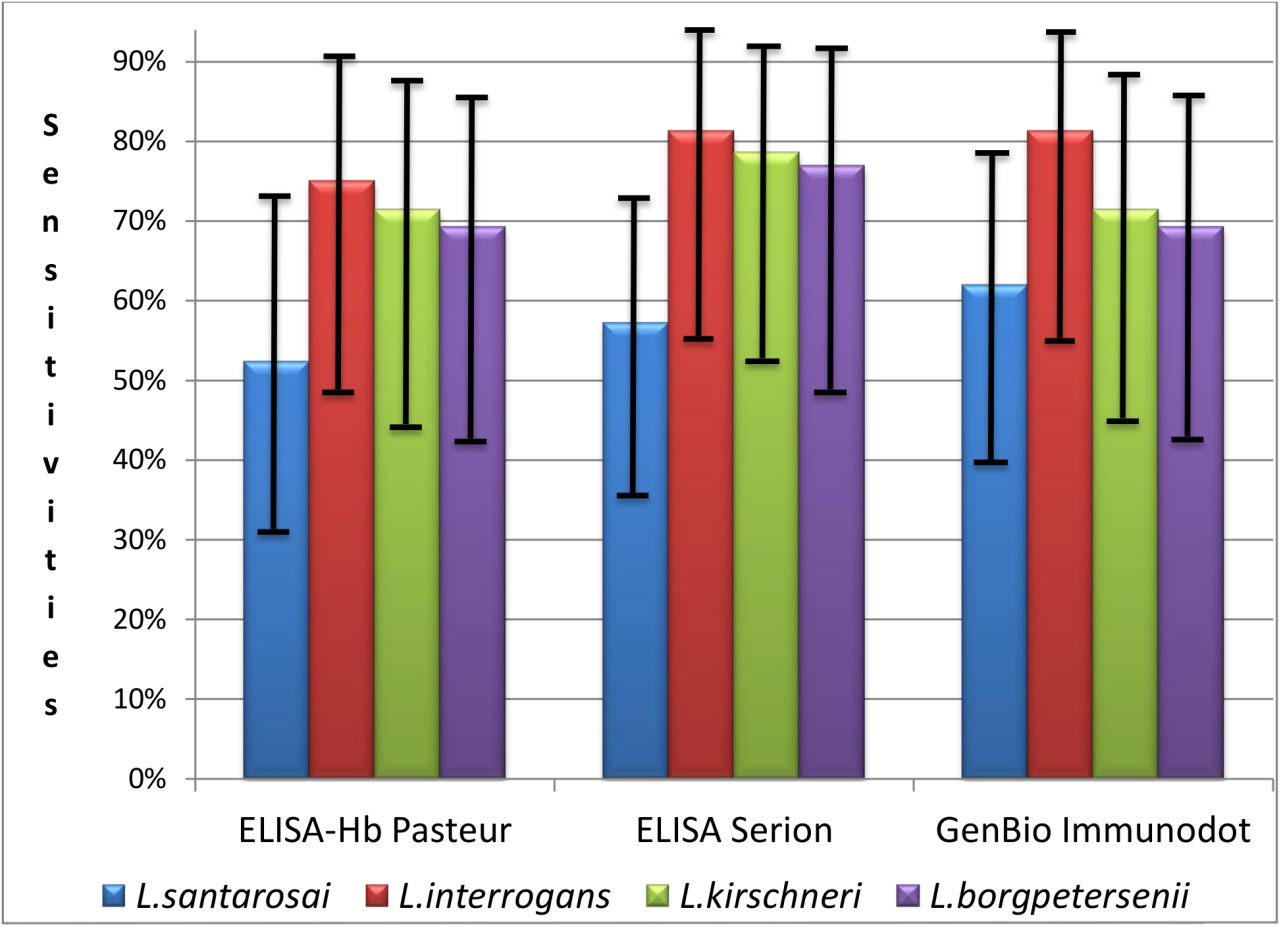
Sensivities and 95% confidence intervals of the 3 serological assays according to genomospecies.

In all, 68 genomospecies were identified, including *L*. *santarosai* (n = 21), *L*. *interrogans* (n = 16), *L*. *kirschneri* (n = 14), *L*. *borgpetersenii* (n = 13), *L*. *noguchii* (n = 2), and *L*. *kmetyi* (n = 2). Sensitivities according to the genomospecies were calculated, and are depicted in Fig 2. Whatever genomospecies studied, the three serological assays gave similar performance. Moreover, *L*. *santarosai* seems to be less reactive with all assays, but this difference was not significant, even with Serion ELISA which exhibit the lowest sensitivity regarding this species (57% vs 81% with *L*. *interrogans*, p = 0.16). Sequence analysis failed to identify the 35 remaining qPCR-positive samples, indicating a low bacterial inoculum (i.e., bacterial loads below the threshold that would give a positive signal by qPCR) in these samples. One may also speculate that the inability to rescue sequences from these samples could be due to false positive results in qPCR (i.e., absence of leptospiral DNA to be amplified), but the high specificity of the qPCR does not support this hypothesis [28].

### Severe cases

Of the 9 severe cases (all associated with *L*. *interrogans*), the median time elapsed from onset of symptoms until obtaining the first serum specimen was 5 days (IQR: 4–5). Unfortunately, because of the very few number of patients included, a statistical analysis was not be performed to relate the disease severity to the results observed.

### Antibiotics data

Data on the type and duration of antibiotic treatment were available for 99 patients. Among them, 52 patients were given antibiotics before blood plasma sample collection. Of these, the lag time between onset of symptoms and diagnosis of leptospirosis was <6 days and ≥6 days for 8 and 44 patients, respectively. Among the latter, 3 were negative for all serological assays, and their median antibiotics treatment duration before blood plasma sampling was 6 days (IQR: 3–9). The 41 other patients with a lag time ≥6 days were positive by at least one of the methods evaluated, and their median treatment duration before blood plasma sampling was 7 days (IQR: 5–10). Of note, precocious antibiotic therapy was not associated with a lower sensitivity, regardless of the serological assay.

## Discussion

This study is the largest consecutive series reported comparing serological tests with qPCR as reference method to define the study cases. Our results show that, with an adapted threshold and precise information regarding the days' post onset of the symptoms, IgM detection tests could be considered reliable diagnostic tools for leptospirosis in an endemic area. The 3 kits evaluated were able to detect at least 6 genomospecies, especially *L*. *interrogans* which is associated with severe leptospirosis [11].

In high prevalence areas, a four-fold (or greater) increase in MAT titer between two consecutive sera is more accurate than a single MAT as unique criteria for leptospirosis case definition [10]. However, most studies comparing serological assays use a single MAT for case definition due to the non-availability of a second serum. Moreover, several studies have shown persistence of IgM antibodies for several months or years after an infection, that may cause false positive results [36–38] In this context, we selected qPCR-positive samples to define our study population. Currently, qPCR is the best available assay for diagnosis confirmation in the early phase of the disease [39,40]. Moreover, according to various studies, detection of leptospiral DNA might be positive until 10 to 15 days after symptoms onset [11,14,41].

The present study focused on assay performances according to DPO. A high completeness in DPO recording allowed us to demonstrate that those 3 assays are efficient as early as 6 DPO, which was consistent with previous studies [42,43]. Interestingly, unlike our results, some studies found no significant link between sensitivity and DPO, possibly because the number of cases was lower, or because DPO was only partially recorded [44,45]. Nevertheless, our findings are in agreement with previously published studies demonstrating that sensitivities of serological assays increased as the diseases progressed [40,46]. Indeed, when considering the latest serum (of a paired sera), a dramatic increase in sensitivity was observed for all tests, suggesting that optimal performance and utilization of serological assays for the diagnosis of leptospirosis require knowledge regarding time of symptom onset. This result could explain the high heterogeneity frequently seen in other comparative studies, as suggested in one meta-analysis [18], and two studies in which sensitivity varied from 4.2% [39] to 96.6% [47] with the same ELISA kit.

Accuracy of our three assays could also be improved by threshold optimization, which allowed us to find these assays give variable performances. Indeed, in spite of threshold optimization, the GenBio immunodot seems to be less sensitive than the 2 ELISAs. Similarly, a number of studies attempted to find the best thresholds for the evaluated assays in order to increase accuracies according to a specific area population [48–50]. This highlights the interest in threshold optimization, particularly in high prevalence areas.

Leptospirosis-free sera selection was conducted mainly to evaluate assays against clinical dengue-like syndromes. Indeed, in tropical and subtropical climates, the clinical manifestations of leptospirosis are often indistinguishables from those of dengue fever, leading to an underestimation of leptospirosis cases during dengue outbreaks [51,52]. All three assays demonstrated high specificity, suggesting that they could be used as diagnostic tools in overlapping endemic areas.

Rapid Diagnostic Tests (RDTs) show interesting characteristics such as rapidity, easy handling [53] and reduced cost [29]. Moreover, they have a good accuracy such as the vertical flow developed by the Pasteur Institute—which uses the same bacteria strain as the ELISA-Hb Pasteur test evaluated here [54]—and as other studies showed [45,53] especially in remote tropical areas. Leptospirosis diagnosis could thus follow the same evolution as other pathologies [55];[56], abandoning latex-based tests (with low specificity) in favor of ELISAs (with good accuracy) or eventually RDTs (with easy handling).

The most important limitation of our study is its retrospective design, thereby precluding calculation of true predictive values. From a methodological point of view, we underline the bias introduced by the selection of cases based on qPCR-positivity, which may contribute to enhance the performances of serological assays since sera were collected with a median of 6 days after the plasma samples.

Our findings highlight the importance of days' post onset of symptoms recording and threshold optimization in high prevalence areas. Indeed, specific anti-*Leptospira* antibodies are seldom detected in acute sera (obtained within 5 days of onset of symptoms), while qPCR seems to be the best currently available diagnostic test in early stage [39]. Conversely, serological tests exhibit very high performances from the 6^th^ DPO. These findings, in agreement with other studies, suggest that, in areas where MAT is not easily available, the combination of serology and qPCR could be the most reliable approach for laboratory confirmation of clinically suspected cases of leptospirosis [46,57–59]. In resource-limited settings, easy handling and cost-effectiveness may be the main decision factors to choose between assays with similar accuracy for routine use.

## Supporting information

S1 Appendix ELISA-Hb Pasteur ROC curve. The nearest threshold form 1, is 1/400.
(TIF)

S2 Appendix ELISA Serion ROC curve. Nearest thresholds from 1 are 17, 25 and 35 UI/mL.
(TIF)

S3 Appendix Immunodot GenBio ROC curve. The nearest threshold form 1, is 1 dot.
(TIF)

S1 Table Supplemental data. (XLSX)

S2 Table STROBE checklist. (DOCX)
