# Supplementary figures and images for: Evaluation of different serological assays for early diagnosis of leptospirosis in Martinique (French West Indies)
Source: PLoS Negl Trop Dis. 2017 Jun 23;11(6):e0005678. doi: 10.1371/journal.pntd.0005678 (PMC5500375; doi:10.1371/journal.pntd.0005678)

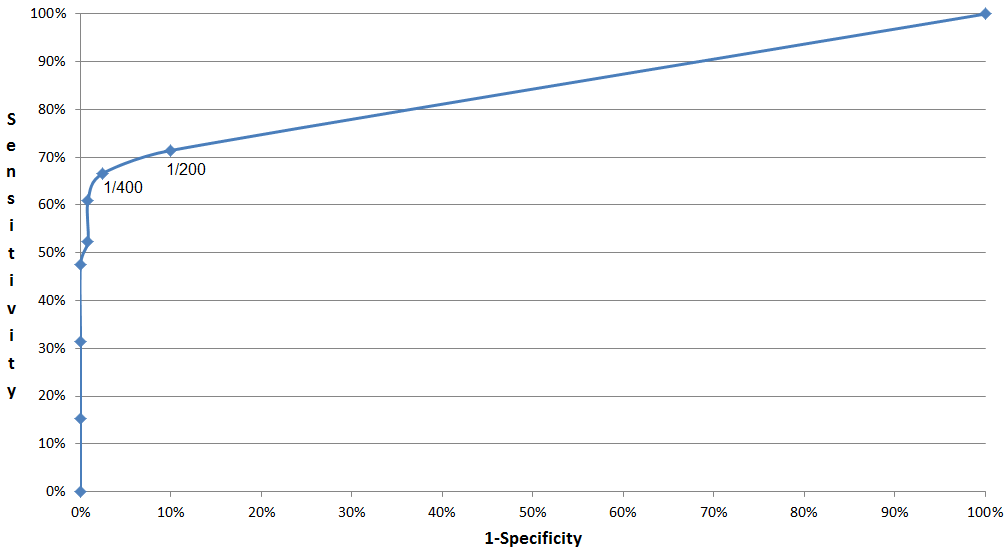

Supplement: S1 Appendix — The nearest threshold form 1, is 1/400. (TIF) [file pntd.0005678.s001.tif]

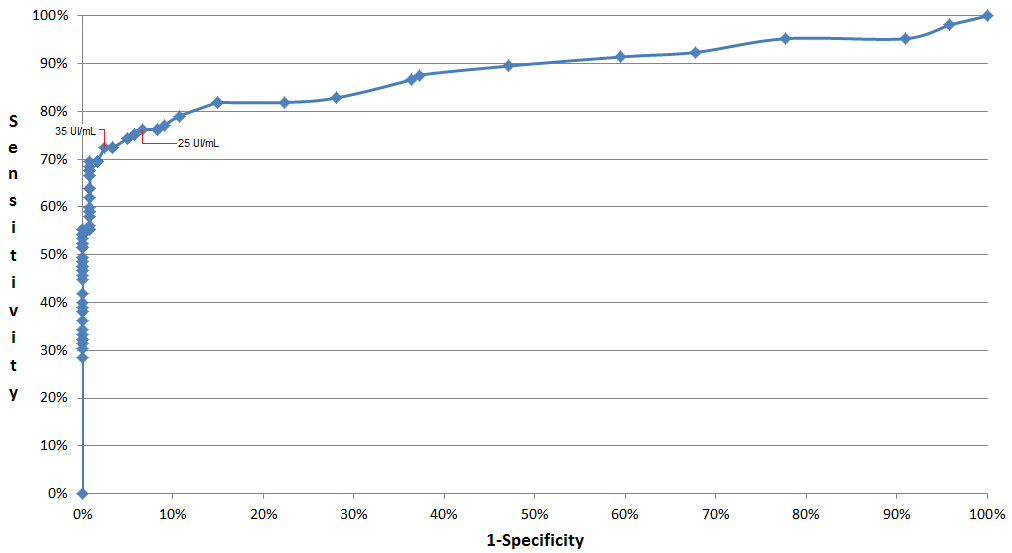

Supplement: S2 Appendix — Nearest thresholds from 1 are 17, 25 and 35 UI/mL. (TIF) [file pntd.0005678.s002.tif]

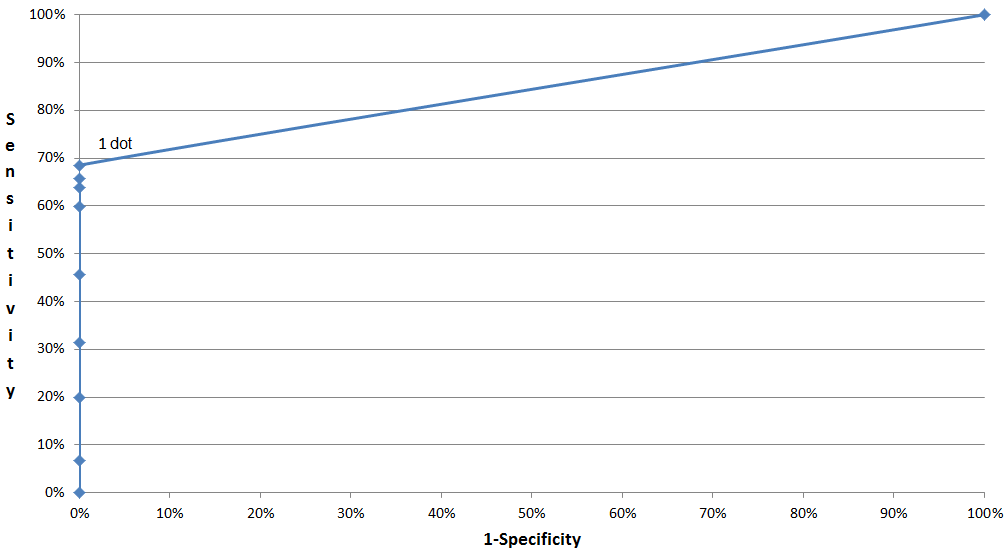

Supplement: S3 Appendix — The nearest threshold form 1, is 1 dot. (TIF) [file pntd.0005678.s003.tif]
